# β3GnT8 Promotes Colorectal Cancer Cells Invasion via CD147/MMP2/Galectin3 Axis

**DOI:** 10.3389/fphys.2018.00588

**Published:** 2018-05-23

**Authors:** Zhi Jiang, Huan Zhang, Chunliang Liu, Jun Yin, Shan Tong, Junxing Lv, Shaohua Wei, Shiliang Wu

**Affiliations:** ^1^Department of Biochemistry and Molecular Biology, School of Medicine, Soochow University, Suzhou, China; ^2^Department of General Surgery, The Second Affiliated Hospital of Soochow University, Suzhou, China; ^3^First People’s Hospital of Changshu City, Changshu Hospital Affiliated to Soochow University, Changshu, China; ^4^Jiangsu Institute of Hematology, The First Affiliated Hospital of Soochow University, Suzhou, China; ^5^Department of General Surgery, The First Affiliated Hospital of Soochow University, Suzhou, China

**Keywords:** β3GnT8, polylactosamine, cell invasion, colorectal cancer, glycosylation

## Abstract

β1,3-N-acetylglucosaminyltransferase (β3GnT8) and β3GnT2 are key enzymes that catalyzes the formation of polylactosamine glycan structures by transferring GlcNAc to tetra-antennary β1-6-branched *N*-glycan and it also has an important effect on the progression of various types of human cancer. They have been reported to participate in tumor invasion and metastasis by regulating the expression of matrix metalloproteinases (MMPs), CD147, and polylactosamine. However, whether β3GnT8 and β3GnT2 play a role in colorectal cancer and, if so, the underlying mechanisms remain unclear. In our study, we detected the expression of β3GnT8, CD147, MMP2, and galectin3 by immunohistochemistry on 90 paraffin-embedded slices. And β3GnT8, CD147, MMP2, and galectin3 were over-expressed in colorectal cancer tissues. We found that overexpression of β3GnT8 and β3GnT2 promoted invasion of colorectal cancer cells, whereas knockdown of β3GnT8 and β3GnT2 inhibited the invasive activity. Mechanistically, β3GnT8 and β3GnT2 regulated the expression of HG-CD147 and the level of polylactosamines in colorectal cancer cells. Together, these results illustrate that the novel role and the molecular mechanism of β3GnT8 and β3GnT2 in promotion of colorectal cancer invasion. These results suggest that the potential use of β3GnT8 as a tumor target for the therapy of colorectal cancer.

## Introduction

Glycans in glycoconjugates including glycoproteins and glycolipids participate in a number of important biological events, including cell–cell interactions, inflammation, and tumor progression (Fuster and Esko, 2005). Poly-*N*-acetyllactosamine (PolyLacNAc), an important glycan structure containing repeats of the *N*-acetyllactosamine (LacNAc) unit (Gal1-4 GlcNAc1–3)*n*, is a fundamental structure of glycans carried on *N*- or *O*-glycans, and is synthesized by β-1,3-*N*-acetylglucosaminyltransferase family (β3GnT) (Ishida et al., 2005). The β3GnT family includes of eight members, β3GnT1 to β3GnT8, which have been identified on the basis of structural similarity to the β1,3-glycosyltransferase conserved motif sequence (Togayachi et al., 2010). When β3GnT8 was first cloned, it was named β3GalT7 and mapped to chromosome 19q13.2 by our laboratory (Huang et al., 2004). β3GalT7 was renamed β3GnT8 on the basis of subsequent enzymatic study (Ishida et al., 2005). Previous studies have reported that β3GnT2 and β3GnT8 are mainly polylactosamine synthases, and suggested β3GnT8 worked as a coordinator with β3GnT2 to elongate the polylactosamine chain of multi-stranded *N*-glycans (Seko and Yamashita, 2005). And the expression of polylactosamine chains was increased by activating intrinsic β3GnT2 activity enhanced by upregulation of β3GnT8 in differentiated HL-60 cells (Seko and Yamashita, 2008). Therefore, β3GnT8 may have an important role in the regulation of the synthesis of polylactosamine.

CD147 is also known as extracellular matrix metalloproteinase inducer (EMMPRIN), which is a target glycoprotein of β3GnT8. And the expression of CD147 was at high levels on many human tumor cells (Ellis et al., 1989; Polette et al., 1997). CD147 has two forms, low glycosylated (LG)-CD147 (∼32 kDa) form and high glycosylated (HG)-CD147 form (∼40–60 kDa). During the malignant transformation, the alteration of CD147 *N*-glycosylation has been demonstrated affected CD147 function (Bai et al., 2014). Previous studies have reported that CD147 deglycosylation induced by tunicamycin could inhibit the expression and secretion of matrix metalloproteinases (MMPs) (Sun and Hemler, 2001). The high polylactosamine content resulted in the elevated the expression of HG-CD147, and CD147 which is a major carrier of β1,6-branched polylactosamines was up-regulated on cancer cells and promoted tumor progression (Tang et al., 2004). Blocking CD147 or CD147-knockdown could induce cell apoptosis of colorectal cancer cells and delayed tumor growth (Baba et al., 2008; Ismail et al., 2016), which suggested targeting CD147 could be used as a potential strategy for colorectal cancer therapy.

Previously studies have reported that galectin-3 was a substrate for MMPs, and was cleaved between Ala62-Tyr63 by active MMP-2 and MMP-9 to form a 22 kDa band (Ochieng et al., 1994; Nangia-Makker et al., 2007). PolyLacNAc was the most preferred ligands for galectin-3 (Sparrow et al., 1987). For example, LAMP1 carries significantly higher levels of PolyLacNAc, and has high affinity ligands for galectin-3 on tumor cell surface (Krishnan et al., 2005).

In previous study of our group, we have found that the positive relationship between β3GnT8 expression and HG-CD147 in the colorectal cancer cell lines, and the level of β3GnT8 was positive correlation with metastatic potential of colorectal cancer cell lines (Ni et al., 2014). Based on our previous study, we next further investigated the role and mechanism of β3GnT8 in colorectal cancer. In the current study, we detected the expression of β3GnT8, CD147, galectin3, and MMP2 in human colorectal cancer tissues and its adjacent paracancer tissues. We overexpressed and knocked down of β3GnT8 in colorectal cancer cell lines to dissect the effect of β3GnT8 on colorectal cancer cells invasion. Moreover, we further elucidated the role of β3GnT8 in regulation of polylactosamines synthesis which related with MMPs and galectin-3 expression.

## Materials and Methods

### Cell Culture and Cell Transfection

The human CRC cell lines SW620, LS174T, and LoVo were cultured in RPMI-1640 (Gibco, Life Technologies) supplemented with 10% inactivated fetal bovine serum (Gibco, Life Technologies). All cell lines were cultured in a humidified atmosphere with 5% CO_2_ at 37°C. The pEX-2-C1 (Mock), pEX-2-β3GnT8, and pEX-2-β3GnT2 plasmids were constructed as previously described (Liu et al., 2014). The pSilenCircle-negative control (NC), pSilenCircle-β3GnT8 (si-β3GnT8), and pSilenCircle-β3GnT2 (si-β3GnT2) plasmids was established by GenePharma (Suzhou, China). Cells were collected 48 h for assays after transfection with Lipofectamine 2000 reagent (Invitrogen, Carlsbad, CA, United States).

### Immunohistochemistry (IHC) Staining

Tissue microarray slides were obtained from Outdo Biotech (Shanghai, China), which contained 90 pairs of adjacent paracancer tissues and colorectal cancer tissues. The slides were stained with primary antibodies against β3GnT8, CD147 (Santa Cruz Biotechnology, Dallas, TX, United States), galectin3 (Abcam, Cambridge, MA, United States), MMP-2 (Abcam), β3GnT2 (Santa Cruz Biotechnology), and HRP-conjugated anti-rabbit IgG, or anti-mouse IgG secondary antibody (Abcam). The protein expression was detected by DAB horseradish peroxidase color development kit (Beyotime, Haimen, China). The slides were evaluated by the staining intensity and positive cells percentage as follows: staining intensity, 0(no), 1(weak), 2(moderate), 3(strong), and positive cells percentage, 0(<1%), 1(1–33%), 2(34–66%), 3(67–100%). The final grade of target protein expression was calculated by plus the score of staining intensity and the score of positive cells percentage: 0(0), 1+(1–2), 2+(3–4), and 3+(5–6). The expression scores of β3GnT8, CD147, galectin3 and MMP-2 were provided in Supplementary Data Sheet 1 (Supplementary Tables 1–4).

### Quantitative Real-Time PCR Analysis

The mRNA of CRC cell lines was isolated and reverse-transcripted to cDNAs by the reverse transcription kit (Invitrogen). Then the cDNAs was used for quantitative PCR analysis using SYBR Green Master Mix Kit (Toyobo, Osaka, Japan) and an ABI detection system (Applied Biosystems, Foster City, CA, United States). The PCR primers were as follows: GAPDH forward, 5′-AGAAGGCTGGGGCTCATTTG-3′ and reverse, 5′-AGGGGCCATCCACAGTCTTC-3′; β3GnT8 forward, 5′-GTCGCTACAGTGACCTGCTG-3′ and reverse, 5′-GTCTTTGAGCGTCTGGTTGA-3′; β3GnT2 forward, 5′-ATACTGGAACCGAGAGCAAG-3′ and reverse, 5′-TCAGGTTCGCAGTAGTTCAG-3′; *CD147* forward, 5′-ACCGTAGAAGACCTTGGCTC-3′ and reverse, 5′-CGTCGGAGTCCACCTTGAAC-3′; *MMP2* forward, 5′-TATGGCTTCTGCCCTGAGAC-3′ and reverse, 5′-CACACCACA TCTTTCCGTCA-3′; *Galectin3* forward, 5′-GTGCCTCGCATGCTGATAAC-3′ and reverse, 5′-ACACATGTAAGTGCAAACAATGACT-3′. The relative expression data were calculated by 2^-ΔΔ*C*_t_^ method using GAPDH as internal control.

### Western Blot Analysis

Total proteins were extracted from CRC cell lines using RIPA lysis buffer containing 1 mM PMSF (Pierce, Rockford, IL, United States) and quantified by BCA Protein Assay Kit (Pierce, Rockford, IL, United States). Proteins were separated on SDS-PAGE and transferred to NC membranes (EMD Millipore, Billerica, MA, United States). After blocking with 5% skimmed milk, the membranes were incubated with different antibodies at 4°C overnight. Following three washes in TBS containing Tween-20, the membranes were incubated at room temperature for 2 h with the secondary antibody. The protein bands were detected by ECL western blot kit (GE Healthcare Life Sciences, Shanghai, China). The primary antibodies used were as follows: anti-β3GnT8 (produced by our laboratory) (Jiang et al., 2010), anti-CD147, anti-β3GnT2, anti-galectin3, anti-GAPDH (Abcam), or β-actin (Abcam).

### Wound Healing Assay

SW620 cells (4 × 10^5^ cells per well) were seeded into a 12-well plate and incubated overnight. A wound was created by scraping monolayer cells with a sterile pipette tip. Cell motility was examined using a light microscope. The photographs were taken immediately (0) and 24 h after wounding. The resulting experiments were analyzed by the ImageJ software (National Institutes of Health, Bethesda, MD, United States). The area of each wound was calculated at each time point.

### Flow Cytometric Analysis

The polylactosamine structures of cell-surface glycoproteins were detected using biotin-labeled *Solanum lycopersicum* (tomato) agglutinin lectin (LEA; Sigma-Aldrich; Merck KGaA, Darmstadt, Germany). Cells were collected, washed three times with PBS and adjusted to 3 × 10^6^ cells/ml. Then the cells were stained with 10 μg/ml LEA at 37°C for 1 h. Washed the stained cells three times with PBST (PBS containing 0.05% Tween-20) and then stained cells with 10 μg/ml PE-conjugated streptavidin (Sigma-Aldrich; Merck KGaA) at 37°C for 1 h. The cells were washed and measured for the fluorescence intensity by BD Calibur flow cytometer. The data was analyzed with Cell Quest software (BD Biosciences, United States).

### MALDI-TOF/TOF-MS Analysis

Total proteins were extracted from CRC cell lines and quantified by BCA Protein Assay Kit. Proteins (2 mg) were used for MALDI-TOF/TOF-MS analysis. The assay procedure was performed as described previously (Yang et al., 2015). *N*-glycans were analyzed according to method reported (Ceroni et al., 2008).

### Statistical Analysis

Data are presented as means ± standard deviation (SD). The statistical analysis was done by Student’s *t*-test using SPSS software (version 22.0, SPSS Inc.). For all analysis, *p* less than 0.05(^∗^) was considered to indicate a statistically significant difference, and *p*-value was indicated as ^∗^*p* < 0.05, ^∗∗^*p* < 0.01, and ^∗∗∗^*p* < 0.001.

## Results

### Expression of β3GnT8 Is Increased in Human Colorectal Cancer Tissues

**FIGURE 1:**
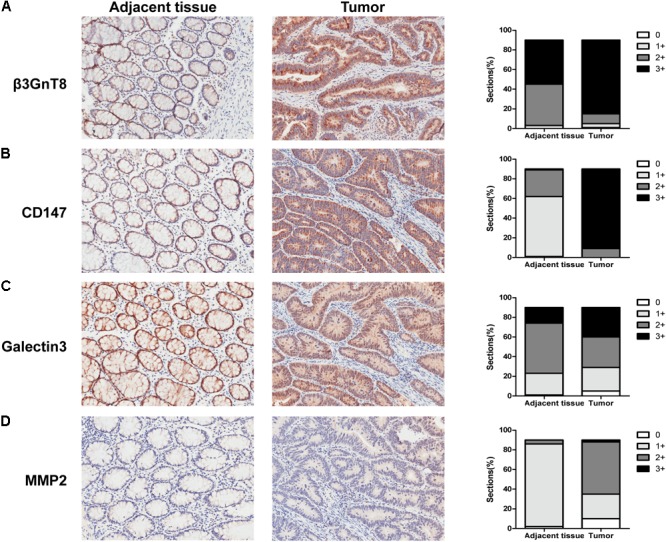
β3GnT8, CD147, galectin3, and MMP2 expressions in colorectal cancer tissues. **(A–D)** Immunohistochemical staining for the expression of β3GnT8, CD147, galectin3, and MMP2 in adjacent paracancer tissues and colorectal cancer tissues. The summary data of immunohistochemical staining score was evaluated by staining intensity and positive cells percentage. Magnification, ×200.

To investigate the effect and correlation of β3GnT8 with the progression of colorectal cancer, we used immunohistochemical staining method to detect the expression of β3GnT8 in 90 pairs of colorectal cancer tissues and its adjacent paracancer tissues (**Table 1**). We found that the expression level of β3GnT8 was higher in colorectal cancer tissues than in adjacent paracancer tissues (**Figure 1A**). Consistent with β3GnT8, the expression of CD147, galectin3, and MMP2 were also up-regulated in colorectal cancer tissues (**Figures 1B–D**). These results suggested that β3GnT8 expression was positively correlated with CD147, galectin3, and MMP2 expression in colorectal cancer tissues. However, the expression of β3GnT2 was decreased in colorectal cancer tissues (Supplementary Figure 1), which was contrary to the β3GnT8 expression. Then the relationship between β3GnT8, CD147, galectin3, MMP2 expression and clinico-pathological features of colorectal cancer was analyzed. However, the expression of β3GnT8 CD147, galectin3, and MMP2 have no correlation with any clinic-pathological factors (**Table 1**).

**Table 1:** Relationship between β3GnT8, CD147, galectin3, MMP2 expression and clinicopathological features of colorectal cancer patients.

Clinico-pathological features	*n*	β3GnT8		*P*	CD147		*P*	Galectin3		*P*	MMP2		*P*
		High	Low		High	Low		High	Low		High	Low	
**Age**													
<60	16	15	1	0.622	15	1	0.031	6	10	0.27	5	11	0.927
≥60	74	64	10		74	0		39	35		24	50	
**Gender**													
Male	45	41	4	0.334	44	1	0.315	25	20	0.292	14	31	0.822
Female	45	38	7		45	0		20	25		15	30	
**Tumor size**													
<5 cm	34	31	3	0.443	33	1	0.197	20	14	0.192	10	24	0.657
≥5 cm	56	48	8		56	0		25	31		19	37	
**7-year survival**													
Yes	47	42	5	0.631	47	0	0.293	27	20	0.14	15	32	0.948
No	43	37	6		42	1		18	25		14	29	
**TNM stage**													
I+II	55	47	8	0.399	55	0	0.207	30	25	0.28	19	36	0.544
III+IV	35	32	3		34	1		15	20		10	25	
**Lymph node metastasis**													
Positive	36	32	4	0.793	35	1	0.218	15	21	0.197	11	25	0.782
Negative	54	47	7		54	0		30	24		18	36	

### β3GnT8 and β3GnT2 Promotes Colorectal Cancer Cell Invasion *in Vitro*

**FIGURE 2:**
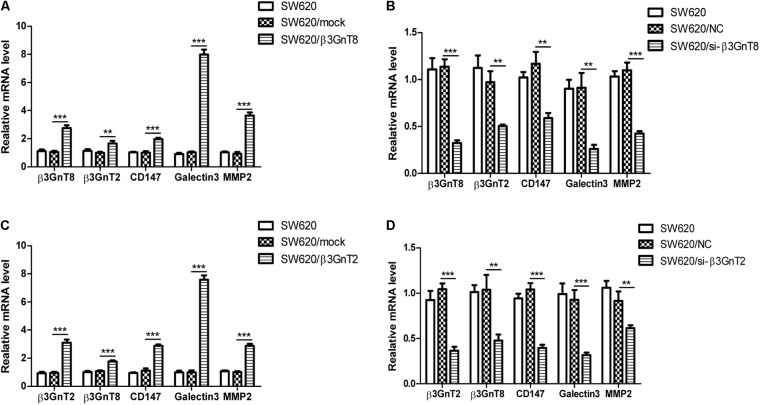
mRNA expression of β3GnT8, β3GnT2, CD147, galectin3, and MMP2 in colorectal cancer cells using quantitative real-time PCR. **(A)** Quantitative RT-PCR analysis of β3GnT8, β3GnT2, galectin3, and MMP2 expression in β3GnT8-overexpressing colon cancer cells. **(B)** Quantitative RT-PCR analysis of β3GnT8, β3GnT2, galectin3, and MMP2 expression in β3GnT8-silenced colorectal cancer cells. **(C)** Quantitative RT-PCR analysis of β3GnT8, β3GnT2, galectin3, and MMP2 expression in β3GnT2-overexpressing colon cancer cells. **(D)** Quantitative RT-PCR analysis of β3GnT8, β3GnT2, galectin3, and MMP2 expression in β3GnT2-silenced colorectal cancer cells. Data are expressed as means ± SD and are representative of three independent experiments. ^∗∗^*p* < 0.01, ^∗∗∗^*p* < 0.001.

**FIGURE 3:**
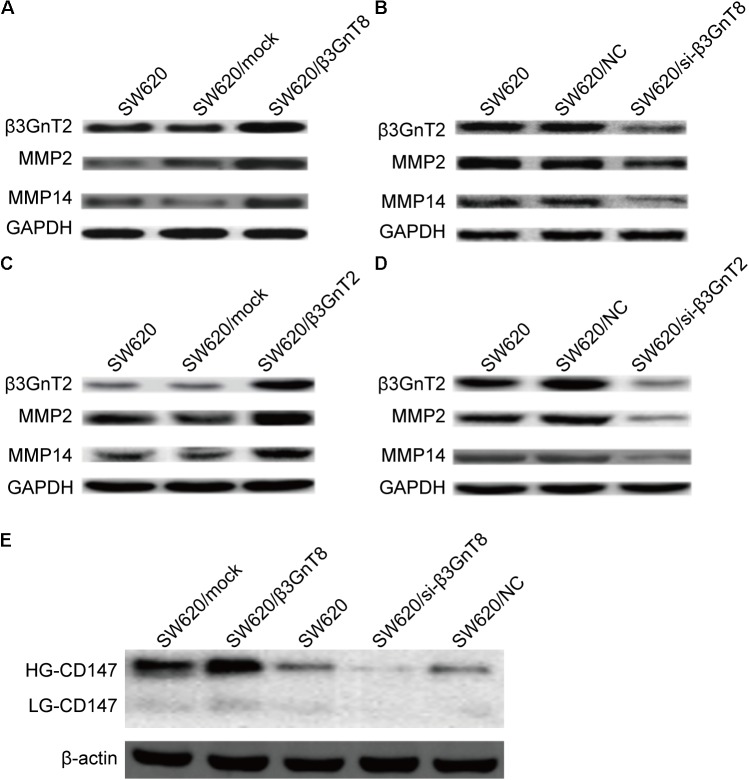
Protein expression of β3GnT2, CD147, MMP2, and MMP14 in colorectal cancer cells. **(A)** Expression of β3GnT2, MMP2, and MMP14 in β3GnT8-overexpressing colon cancer cells by western blot analysis. **(B)** Expression of β3GnT2, MMP2, and MMP14 expression in β3GnT8-silenced colorectal cancer cells by western blot analysis. **(C)** Expression of β3GnT2, MMP2, and MMP14 expression in β3GnT2-overexpressing colon cancer cells by western blot analysis. **(D)** Expression of β3GnT2, MMP2, and MMP14 expression in β3GnT2-silenced colorectal cancer cells by western blot analysis. **(E)** Expression of CD147 in β3GnT8-overexpressing and β3GnT8-silenced colon cancer cells by western blot analysis.

**FIGURE 4:**
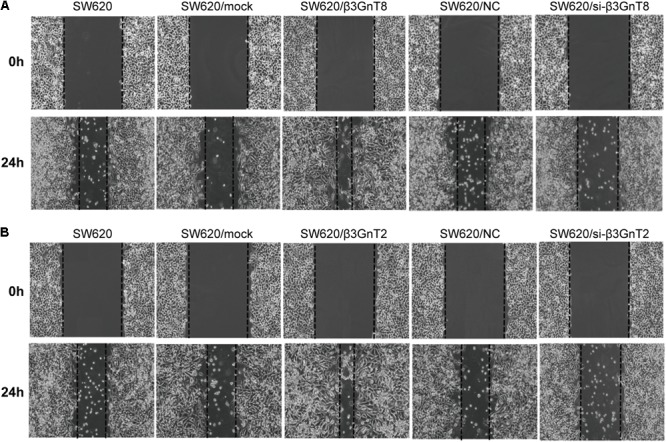
Analysis of cell migration using a wound healing assay. **(A)** SW620 cells were transfected with β3GnT8 plasmid vector and β3GnT8 short interfering RNA vector (si-β3GnT8). Cell motility was examined using a light microscope at 0 and 24 h after wounding. Magnification, ×40. **(B)** SW620 cells were transfected with β3GnT2 plasmid vector and β3GnT2 short interfering RNA vector (si-β3GnT2). Cell motility was examined using a light microscope at 0 and 24 h after wounding. Magnification, ×40.

Based on the previous study of our group (Ni et al., 2014), we choose the SW620 cell line to explore the role of β3GnT8 and β3GnT2. The β3GnT8 over-expression cells and β3GnT8 knockdown cells were successfully constructed (**Figures 2A,B**, **3A,B**). We also constructed β3GnT2 over-expression cells and β3GnT2 knockdown cells (**Figures 2C,D**, **3C,D**). Then we used these cells to perform wound healing assay to deliberated the effect of β3GnT8 on the invasion colorectal cancer cells. The β3GnT8-overexpression promoted cell invasion and β3GnT8-knockdown suppressed cell invasion dramatically after 24 h incubation (**Figure 4A**). We also found the same results in the cells overexpression of β3GnT2 or knockdown of β3GnT2 (**Figure 4B**). These results suggested that β3GnT2 and β3GnT8 were sufficient to promote colorectal cancer invasion *in vitro*, respectively.

### β3GnT8 Promotes Cell Invasion via CD147, Galectin 3, and MMPs Expression

We investigated whether β3GnT8 could affect the expression of CD147, galectin 3, and MMPs. β3GnT8 overexpression significantly increased the mRNA expression of CD147, galectin 3, and MMPs (**Figures 2A,B**). As expected that expression of β3GnT2 also significantly elevated the mRNA levels of CD147, galectin 3, and MMPs in colorectal cancer cells in comparison with the controls (**Figures 2C,D**). The same results were shown in Supplementary Figure 2 for other colorectal cancer cell lines. Then we analyzed the protein levels of those tumor-related genes, and found that both β3GnT8 and β3GnT2 could markedly elevate MMP2 and MMP14 expression (**Figure 3**). Collectively, these results indicated that β3GnT8 promoted cell invasion via enhancing the expression of galectin 3 and MMPs.

### β3GnT8 Promotes Cell Invasion via Enhancing HG-CD147 Glycosylation

We detected the CD147 glycosylation in colorectal cancer cells by western blot analysis. And we found that β3GnT8-overexpression significantly increased the expression of HG-CD147, while β3GnT8-knockdown decreased the levels of HG-CD147 in colorectal cancer cells (**Figure 3E**). However, β3GnT8 almost has no effect on the expression of LG-CD147. These results suggested β3GnT8 could regulation the function of CD147. Therefore, the heterogeneous *N*-glycosylated forms of CD147 may be regulated by β3GnT8 in colorectal cancer cells. Therefore, β3GnT8 promoted the expression of MMP2 though enhancing the HG-CD147 glycosylation.

### β3GnT8 Regulates Polylactosamines Expression in Colorectal Cancer Cells

**FIGURE 5:**
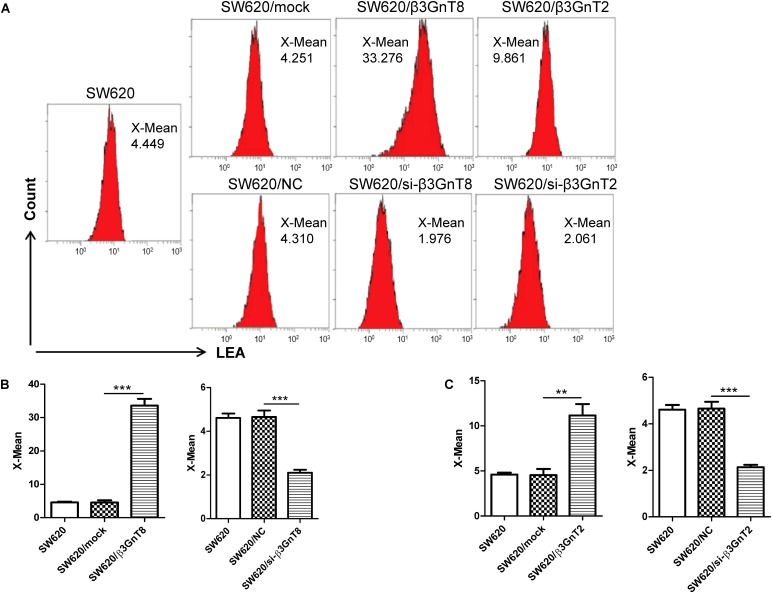
Flow cytometric analysis of polylactosamine expression in colorectal cancer cells. **(A)** Flow cytometric assay was performed to detect the level of polylactosamine expression in β3GnT8 or β3GnT2-overexpressing and β3GnT8 or β3GnT2-silenced SW620 colorectal cancer cells. **(B,C)** Summary data of the X-mean of polylactosamine expression in SW620 colorectal cancer cells. Data are expressed as means ± SD and are representative of three independent experiments. LEA, *Solanum lycopersicum* agglutinin; X-mean, mean intensity of fluorescence. ^∗∗^*p* < 0.01, ^∗∗∗^*p* < 0.001.

The polylactosamine chains on *N*-linked β1,6-branch affect the development of cancer (Huang et al., 2013), we further detected the expression of total polylactosamines in SW620 cells using flow cytometric analysis. We found that of β3GnT8-overexpression upregulated the polylactosamines expression, while β3GnT8-knockdown down-regulated the polylactosamines expression in SW620 cells (**Figures 5A,B**). β3GnT2 performed the same result as β3GnT8 (**Figures 5A,C**). These results suggest that β3GnT8 and β3GnT2 have significant effects on the biosynthesis of polylactosamine chain which affect the expression of MMPs and galectin3.

### β3GnT8 Changes *N*-glycan Patterns in Colorectal Cancer Cells

*N*-glycosylation patterns were aberrant in many cancers, suggesting that the cancer-associated *N*-glycans may be potential tumor biomarkers (Meany and Chan, 2011; Adamczyk et al., 2012). The total *N*-glycans in colorectal cancer cells were profiled by MALDI-TOF/TOF-MS analysis. We found that the number of *N*-glycan structures was 29 in LS174T/mock cells and 28 in LS174T/β3GnT8 cells, but only 19 in LS174T/β3GnT2 cells (Supplementary Figure 3). As shown in Supplementary Figure 4, the number of *N*-glycan structures was 19 in LoVo/NC cells and 17 in LoVo/si-β3GnT8 cells, but 21 in LoVo/si-β3GnT2 cells. Overexpression of β3GnT8 and β3GnT2 only increased the percentage of high-mannose-type in LS174T cells (**Table 2**).

**Table 2:** *N*-glycans types in colorectal cancer cell lines.

Glycan type	Relative proportion (%)					
	LS174T			LoVo		
	Mock	β3GnT8	β3GnT2	NC	si-β3GnT8	si-β3GnT2
High mannose	45.4%	87.1%	68.8%	90.5%	92.5%	89.3%
Complex	32.6%	10.5%	26.2%	7.8%	5.0%	8.9%
Hybrid	14.6%	8.3%	19.1%	7.0%	6.4%	6.9%
Bi-antennary	25.7%	7.6%	20.8%	6.4%	4.2%	7.6%
Tri- and Tetra-antennary	6.9%	3.5%	7.0%	2.6%	0.7%	2.6%
Bisecting GlcNAc	15.7%	6.3%	17.3%	6.4%	4.2%	6.3%
Fucosylation	43.2%	16.1%	36.5%	17.0%	11.2%	18.0%
Sialylation	5.6%	2.4%	4.1%	2.8%	3.1%	2.8%
Lactose	3.6%	2.5%	5.2%	0.0%	0.0%	0.5%

## Discussion

Colorectal cancer is a leading cause of cancer-associated mortality (Siegel et al., 2017). Aberrant glycosylation involved in colorectal cancer progression (de Freitas Junior and Morgado-Diaz, 2016). Previous studies have reported that the β1,6 branches of *N*-glycans are associated with the invasion and metastasis of colorectal cancer and an increase of β1,6 branches on *N*-glycans is commonly observed with malignant transforms. Characteristics of β1,6-branched *N*-glycans are considered hallmarks of colorectal cancer progression (Ishida et al., 2005).

In present study, we showed that the expression of β3GnT8, CD147, galectin3, and MMP2 were significantly higher in colorectal cancer tissues, while the expression of β3GnT2 was decreased in cancer tissues. β3GnT8-overexpression promoted the invasion of colorectal cancer cells, while β3GnT8-knockdown suppressed the ability of cell invasion, these results suggested that β3GnT8 played an important role in the development of colorectal cancer. Our study also found that β3GnT8 can regulate the expression of β3GnT2, CD147, galectin3, and MMPs. We know that not only high expressions of MMPs and CD147 are associated with tumor invasion, but also high expression of tumoral galectin-3 was associated with tumor size and poor differentiation but negatively related to low E-cadherin expression (Huang et al., 2016). Our findings indicated that β3GnT8 could promote colorectal cancer invasion by enhancing the expression of MMPs, CD147, and galectin3. We also know that β3GnT8 and β3GnT2 can form a heterocomplex and the enzymatic activity is enhanced, this suggest that β3GnT2 and β3GnT8 may be cooperatively regulated the polylactosamine chains elongation (Seko and Yamashita, 2005). Previous study also suggested that upregulation of β3GnT8 could enhance β3GnT2 activity to increase the expression of polylactosamines in differentiated HL-60 cells (Seko and Yamashita, 2008).

β3GnT8 was expressed highly in gastric cancer and regulated the metastasis of gastric cancer cells via modulating the polylactosamines of CD147 (Shen et al., 2017). Our lab has also demonstrated that β3GnT8 may affect the signaling pathway of CD147 (Jiang et al., 2014). CD147 has a high expression on surface of tumor cells (Sameshima et al., 2000; Pan et al., 2012; Zhu et al., 2013). Also, CD147 has the high-glycosylated forms, and plays key roles in metastasis of tumors. The HG-CD147 could stimulate tumor cells to produce MMPs (Jiang et al., 2001). Therefore, we investigated whether β3GnT8 affect the glycosylation of CD147 in colorectal cancer cells. And we demonstrated that overexpression of β3GnT8 increased the expression of HG-CD147 in colorectal cancer cells, and knockdown of β3GnT8 reduced HG-CD147 expression, suggesting that β3GnT8 might regulate the expression of MMP2 through altering CD147 glycosylation in colorectal cancer. And our lab has also demonstrated that the polylactosamines level in CD147 was regulated by β3GnT8 via IP assay (Shen et al., 2017). These studies indicated β3GnT8 affect the tumor development through MMPs expression which could be regulated by CD147 glycosylation (Sameshima et al., 2000).

β3GnT8 was reported participated in the regulation of polylactosamines synthesis on β1,6-branched *N*-glycans (Ishida et al., 2005; Seko and Yamashita, 2005). And global changes in protein glycosylation are associated with cancer (Kim et al., 2009). We found β3GnT8-overexpression increased and β3GnT8-knockdown reduced the expression of polylactosamines and glycopattern abundance in colorectal cancer cells, Moreover, the expression of MMPs and galectin3, which was regulated by polylactosamines, was also positively correlated with β3GnT8. Our results suggested that β3GnT8 could change CD147 glycosylation and global protein glycosylation.

Based on our previous study, we found β3GnT8 expression was increased in colorectal cancer tissues, and the β3GnT8 expression was positively correlated with CD147, galectin3, and MMP2 expression in colorectal cancer tissues and cell lines in this study. Therefore, β3GnT8 may promotes colorectal cancer invasion via enhancing the expression of MMPs, CD147, and galectin3. And we confirmed that β3GnT8 promoted the invasion of colorectal cancer cells through increasing the expression of HG-CD147. The expression of β3GnT8 and β3GnT2 was not positively correlated in clinical colorectal cancer tissues, while β3GnT8 worked as a coordinator with β3GnT2 to regulate the expression of polylactosamine and MMPs *in vitro*. Our findings demonstrated that β3GnT8 plays an important role in the progression of colorectal cancer, suggesting that the potential use of β3GnT8 as a tumor target for the prevention of colorectal cancer invasion.

## Ethics Statement

This study was approved by the ethics committee of the Soochow University. Informed consent was obtained from all patients in the study.

## Author Contributions

ZJ and SlW designed the study and wrote the manuscript. ZJ, HZ, ST, JL, and JY performed the experiments. ZJ, CL, and ShW performed the statistical analyses and critiqued the manuscript. All authors read and approved the final manuscript

## Conflict of Interest Statement

The authors declare that the research was conducted in the absence of any commercial or financial relationships that could be construed as a potential conflict of interest. The reviewer XL and handling Editor declared their shared affiliation.
