# Supplementary material for: β3GnT8 Promotes Colorectal Cancer Cells Invasion via CD147/MMP2/Galectin3 Axis
Source: Front Physiol. 2018 May 23;9:588. doi: 10.3389/fphys.2018.00588 (PMC5974207; doi:10.3389/fphys.2018.00588)
Supplement: Supplementary file 2 [file Image_1.PDF]

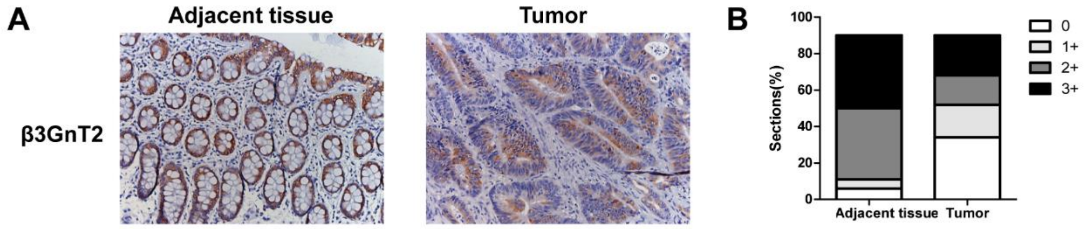

sFigure 1 β3GnT2 expressions in colorectal cancer tissues.

(A, B) Immunohistochemical staining for the expression of β3GnT2 in colorectal cancer tissues and the adjacent paracancer tissues, which was evaluated according to the percentage of positive cells and the staining intensity. Magnification, ×200.

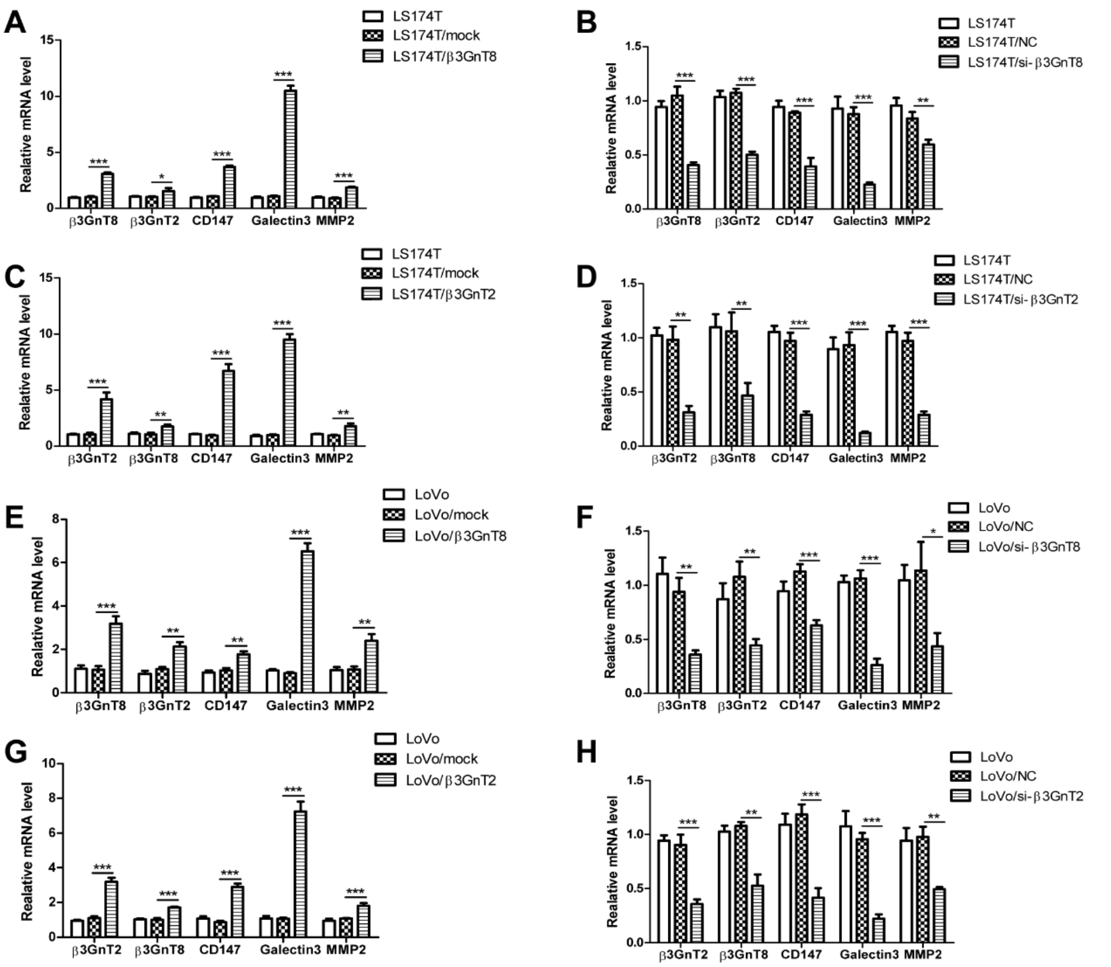

sFigure 2 mRNA expression of β3GnT8, β3GnT2, CD147, Galectin3 and MMP2 in colorectal cancer cells using quantitative real-time PCR.

(A) Quantitative RT-PCR analysis of β3GnT8, β3GnT2, Galectin3 and MMP2 expression in β3GnT8-overexpressing LS174T colon cancer cells. (B) Quantitative RT-PCR analysis of β3GnT8, β3GnT2, Galectin3 and MMP2 expression in β3GnT8-silenced LS174T colorectal cancer cells. (C) Quantitative RT-PCR analysis of β3GnT8, β3GnT2, Galectin3 and MMP2 expression in β3GnT2-overexpressing LS174T colon cancer cells. (D) Quantitative RT-PCR analysis of β3GnT8, β3GnT2, Galectin3 and MMP2 expression in β3GnT2-silenced LS174T colorectal cancer cells. (E) Quantitative RT-PCR analysis of β3GnT8, β3GnT2, Galectin3 and MMP2 expression in β3GnT8-overexpressing LoVo colon cancer cells. (F) Quantitative RT-PCR analysis of β3GnT8, β3GnT2, Galectin3 and MMP2 expression in β3GnT8-silenced LoVo colorectal cancer cells. (G) Quantitative RT-PCR analysis of β3GnT8, β3GnT2, Galectin3 and MMP2 expression in β3GnT2-overexpressing LoVo colon cancer cells. (H) Quantitative RT-PCR analysis of β3GnT8, β3GnT2, Galectin3 and MMP2 expression in β3GnT2-silenced LoVo colorectal cancer cells. Data are representative of three independent experiments and presented as means ± SD; $*p < 0.05$, $**p < 0.01$, $***p < 0.001$.

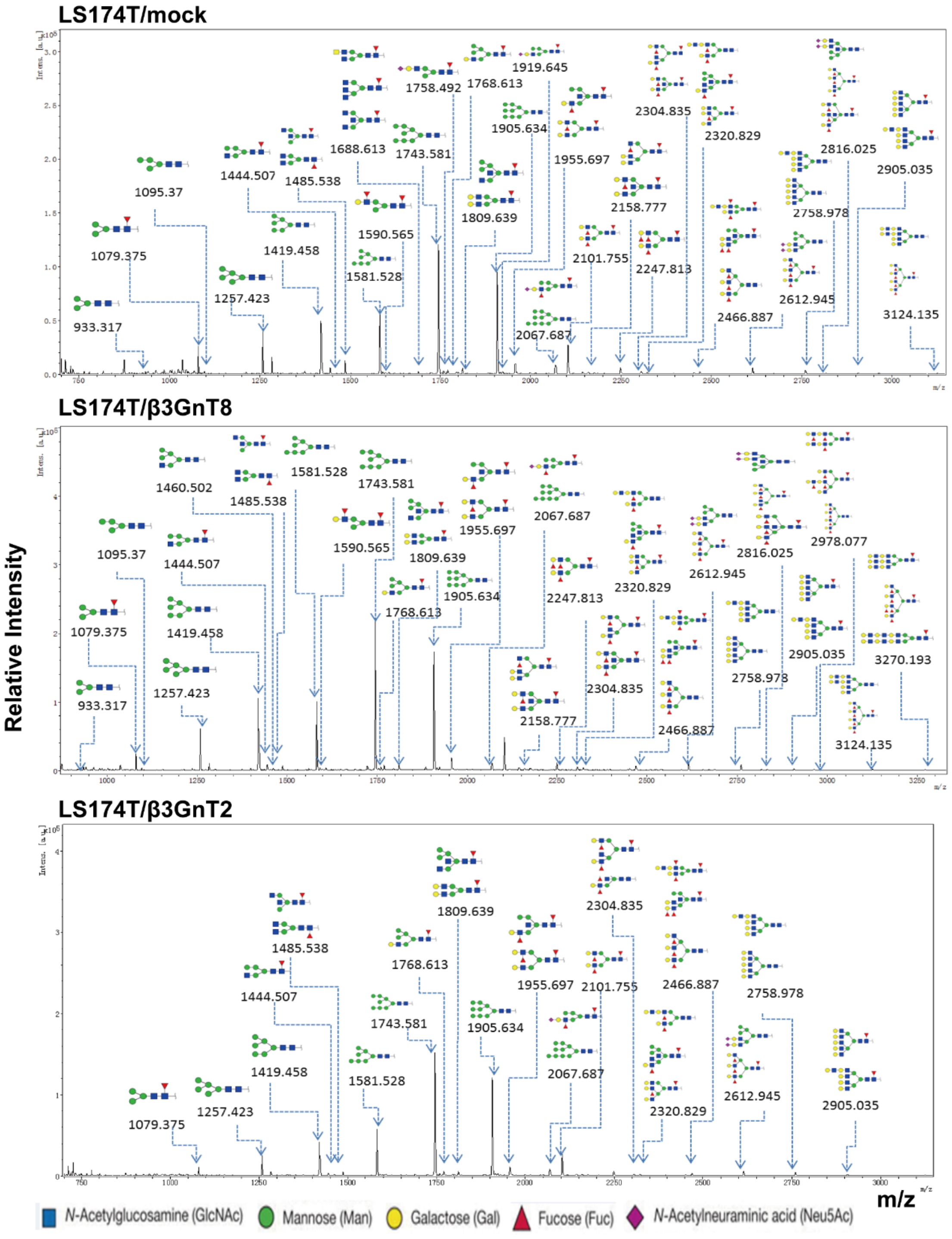

sFigure 3 MALDI-TOF/TOF-MS/MS analysis of *N*-glycan precursor ions in LS174T colorectal cancer cells.

MS/MS analysis of precursor ions was performed and cleavages were obtained, including B, Y, C, and Z glycosidic cleavages and A and X cross-ring cleavages. Structures of cleavage ions and m/z values of LS174T/mock, LS174T/β3GnT8 and LS174T/β3GnT2 are shown.

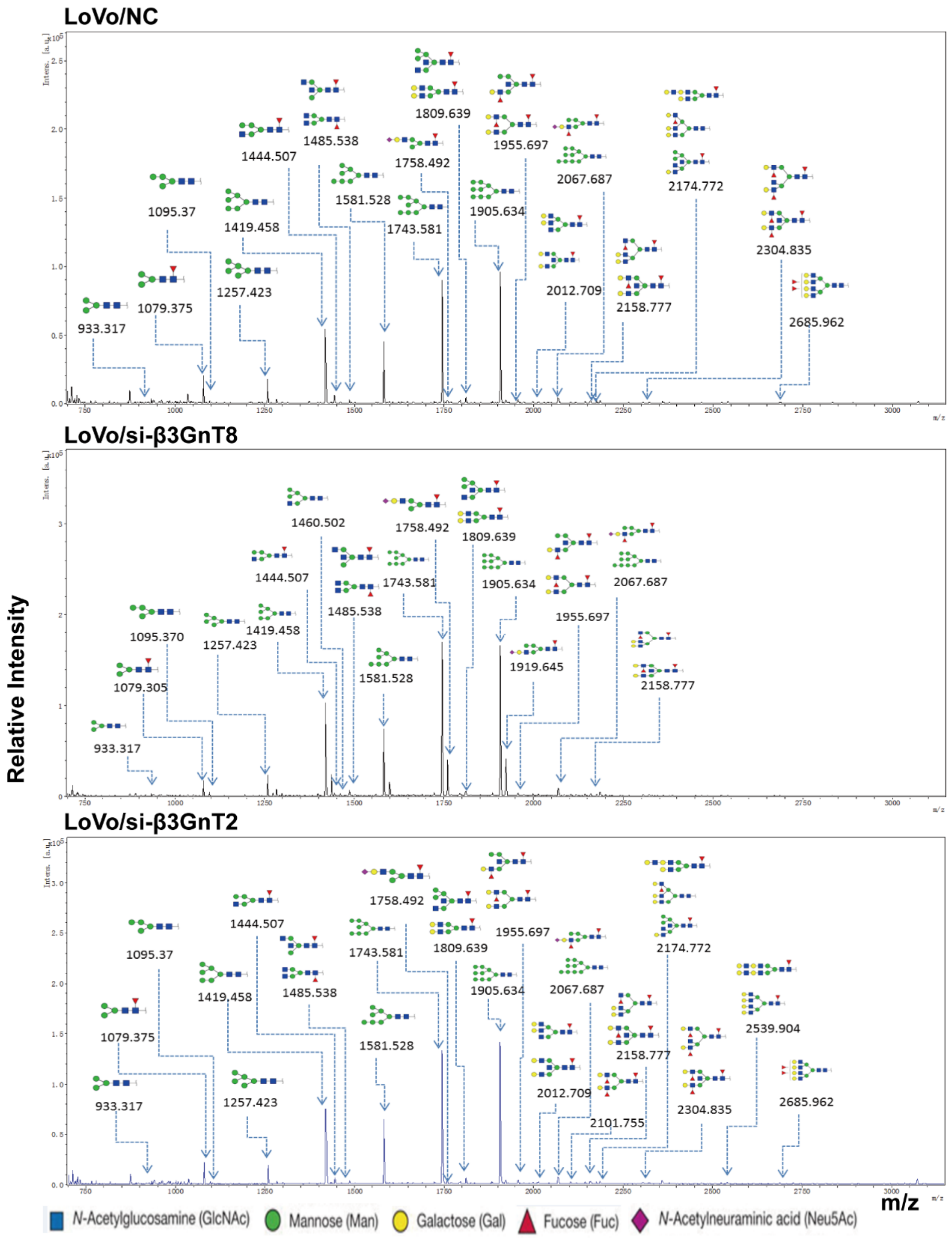

sFigure 4 MALDI-TOF/TOF-MS/MS analysis of *N*-glycan precursor ions in LoVo colorectal cancer cells.

MS/MS analysis of precursor ions was performed and cleavages were obtained, including B, Y, C, and Z glycosidic cleavages and A and X cross-ring cleavages. Structures of cleavage ions and m/z values of LoVo/NC, LoVo/si-β3GnT8 and LoVo/si-β3GnT2 are shown.
